# Antiviral activity of *Rumex crispus* extract and its bioactive constituent emodin against human coronavirus OC43

**DOI:** 10.1371/journal.pone.0362181

**Published:** 2026-11-05

**Authors:** Seungju Cho, Jang Hoon Kim, Siyun Lee, Chunghyeon Lee, Yong-Goo Kim, Eunji Cho, Jayhyun Park, Junsoo Park

**Affiliations:** 1 Division of Biological Science and Technology, Yonsei University, Wonju, Republic of Korea; 2 Department of Herbal Crop Research, National Institute of Horticultural & Herbal Science, Rural Development Administration, Eumsung, Chungbuk, Republic of Korea; 3 Analysis & Evaluation department, Korea Mine Rehabilitation and Mineral Resources Corporation, Wonju, Korea; 4 Department of Analysis and Assessment, Korea Mine Rehabilitation and Mineral Resources Corporation, Wonju, Korea; University of Bergen: Universitetet i Bergen, NORWAY

## Abstract

Coronaviruses are responsible for diseases ranging from mild respiratory symptoms to severe infections such as COVID-19. Despite the availability of vaccines and therapeutic agents, the emergence of new variants and drug-resistant strains highlights the need for additional antiviral strategies. In this study, *Rumex crispus* was investigated as a potential source of antiviral compounds. The ethanol (EtOH) extract of *R. crispus* roots was prepared and subsequently fractionated into n-hexane (n-hex), ethyl acetate (EA), and aqueous fractions. Among these, the n-hexane and EA fractions exhibited significant antiviral activity against human coronavirus OC43, as demonstrated by Western blot, qRT-PCR, and plaque assays. Chemical analysis of the extract by HPLC led to the identification of epicatechin, naphthalene derivatives, and anthraquinones. Notably, emodin, an anthraquinone, showed pronounced antiviral activity, and was found to be enriched in the active n-hexane and EA fractions. These findings suggest that emodin may contribute to the antiviral activity of *R. crispus* extract, although the potential involvement of other bioactive constituents cannot be excluded.

## Introduction

Coronaviruses are a diverse group of viruses that can cause a wide range of diseases, from the common cold to severe respiratory illnesses such as COVID-19 [1,2]. They affect not only humans but also animals, with animal coronaviruses responsible for conditions such as feline infectious peritonitis in cats and porcine epidemic diarrhea in pigs [3–6]. In addition to SARS-CoV-2, which caused the recent COVID-19 pandemic, several human coronaviruses, including HCoV-OC43, HCoV-229E, HCoV-NL63, and HCoV-HKU1, are responsible for a substantial proportion of common cold infections [7,8]. Among these viruses, HCoV-OC43 has attracted considerable attention because it belongs to the genus *Betacoronavirus*, which also includes SARS-CoV-2 and MERS-CoV [9]. Due to its phylogenetic relationship with these highly pathogenic coronaviruses and its relatively low pathogenicity, HCoV-OC43 has been used as a surrogate model for the initial screening and evaluation of potential antiviral agents [10]. Therefore, investigating antiviral compounds against HCoV-OC43 may provide valuable insights into the development of therapeutic candidates targeting human coronaviruses, including SARS-CoV-2 [11].

Coronaviruses possess a positive-sense, single-stranded RNA genome and can undergo genetic changes that contribute to the emergence of new variants, potentially affecting viral transmissibility, pathogenicity, and susceptibility to antiviral treatments [12,13]. Therefore, continued efforts to identify antiviral agents with diverse mechanisms of action are necessary to combat existing and emerging coronavirus infections. To date, plant phytochemicals have provided various approaches to inhibit coronavirus infection, and these antiviral phytochemicals may serve as alternative strategies for treating coronavirus-related diseases [14–16]. *Rumex crispus* L., commonly known as “curly dock,” is named for its characteristically wavy and curly leaves. *This species is widely distributed across Europe, Asia, and North Africa* [17,18]. Although it is considered an invasive species in some regions, the young leaves of *R. crispus* are consumed as a vegetable (e.g., in salads) and have also been used in traditional folk medicine to treat various ailments [18–20]. Extracts of *R. crispus* contain various bioactive compounds, including anthraquinones, which have been reported to exhibit anti-inflammatory, antioxidant, and anticancer activities [21–23].

Although emodin has previously been reported to exhibit antiviral activity against coronaviruses, the antiviral potential of Rumex crispus root extracts and their fractions remains poorly understood [24,25]. In this study, we evaluated the antiviral activity of *R. crispus* root extracts and solvent fractions against HCoV-OC43. Furthermore, we performed phytochemical analysis to identify bioactive constituents associated with the observed antiviral activity. Our findings demonstrate the anti-coronavirus activity of *R. crispus* root fractions and identify emodin as one of the compounds potentially contributing to this effect.

## Materials and methods

### General experimental procedures

Nuclear magnetic resonance (NMR) spectra were recorded at 300 MHz for ¹H and, where measured, 75 MHz for ^13^C using methanol-d₄ or DMSO-d_6_ as the solvent, as indicated in the corresponding Supporting Information captions. Additional ^1^H NMR spectra of emodin (12) and citreorosein (9) were recorded on an AVANCE III-400 spectrometer (Bruker, Billerica, MA, USA) at 400 MHz using DMSO-d_6_ as the solvent. Analytical thin-layer chromatography (TLC) was performed on precoated silica gel phase and C-18 phase (Merck, Rahway, NJ, USA) plates, respectively. Column chromatography was performed using silica gel (60 Å, 230–400 mesh ASTM; Merck, Germany) and C-18 silica gel (ODS-A, S-75 µm; YMC, Kyoto, Japan).

### Plant material

The roots of *R. crispus* were purchased from a traditional herbal medicine store in the Republic of Korea in August 2023 and authenticated by Dr. J.H. Kim. A voucher specimen (RC 23) has been deposited at the herbarium of the Department of Herbal Crop Research, National Institute of Horticultural and Herbal Science, Republic of Korea.

### Extraction of the roots of *R. crispus L*. and isolation of compounds

The roots of *R. crispus* (4 kg) were extracted three times with 99% ethanol (30 L) in a double boiler at 60 ℃. The filtered solvent was evaporated under reduced pressure to obtain an ethanol extract (350 g). The condensed extract was suspended in 8 L of water and then sequentially fractionated into n-hexane (30 g), ethyl acetate (EA, 38 g), and water layers (262 g), respectively. The n-hexane fraction was subjected to silica gel column chromatography using a gradient solvent system of n-hexane and EA (10:0–1:4) to yield 15 fractions (RCh1–RCh15). The RCh7 fraction was chromatographed by C-18 column chromatography using a gradient solvent system of distilled water and methanol (1:0–1:5) to yield two compounds, 12 (450 mg) and 13 (320 mg), and three fractions (RCh71–RCh74). Compound 11 (37 mg) was purified from RCh43 using C-18 column chromatography with an isocratic solvent system of methanol and distilled water (3:1). The RCh12 fraction was separated by C-18 column chromatography using a gradient solvent system of distilled water and methanol (1:0–1:2) to yield two compounds, 9 (25 mg) and 10 (14 mg). The EA fraction was subjected to silica gel column chromatography using a gradient solvent system of chloroform and methanol (20:1–1:3) to obtain twelve fractions (ECe1–ECe12). The ECe4 fraction was chromatographed by C-18 column chromatography using a gradient solvent system of distilled water and methanol (1:0–1:3) to yield two compounds, 8 (54 mg) and 7 (47 mg), and five fractions (ECe41–ECe45). ECe43 was further purified by C-18 column chromatography with an isocratic solvent system to yield compound 6 (39 mg). The ECe7 fraction was subjected to C-18 column chromatography using a gradient solvent system of chloroform and methanol (15:0–1:1) to obtain seven fractions (ECe71–ECe77). The ECe74 fraction was purified by C-18 column chromatography using an isocratic solvent system of methanol and distilled water (1.5:1) to yield three compounds, 5 (25 mg), 4 (21 mg), and 3 (16 mg). The ECe10 fraction was subjected to silica gel column chromatography using a gradient solvent system of chloroform and methanol (10:1–1:3) to obtain five fractions (ECe101–ECe105). Two compounds, 1 (21 mg) and 2 (14 mg), were purified from ECe103 by C-18 column chromatography using a gradient solvent system of methanol and distilled water (0.5:1–1:1).

### HPLC analysis

The extract and thirteen compounds (1–13) were analyzed using an Agilent 1260 HPLC system (Agilent Technologies, Santa Clara, CA, USA). The mobile phase consisted of distilled water containing 0.1% formic acid and acetonitrile containing 0.1% formic acid (A), with the following gradient system: 5% A at 0 min, 5–55% A from 0.1 to 30 min, 55.1–100% A from 30 to 37 min, 100% A from 37 to 40 min, and 5% A from 40.1 to 45 min.

The column used was an Agilent Eclipse Plus C18 (5 µm, 4.6 × 250 mm). The detection wavelength, column temperature, and flow rate were set at 254 nm, 25 ℃, and 1.0 mL/min, respectively. For qualitative and quantitative analysis, the concentrations of the extract and three fractions were prepared at 5 mg/mL. The calibration curve for quantitative analysis of compound 12 was established at concentrations ranging from 3.1 to 100 µg/mL.

**Infection of coronavirus**Human coronavirus OC43 (HCoV-OC43) was obtained from the American Type Culture Collection (ATCC, Rockville, MD, USA), and rhabdomyosarcoma (RD) cells were provided by the Korean Cell Line Bank (Seoul, Korea). RD cells were cultured in Dulbecco’s Modified Eagle’s Medium (DMEM; Welgene, Seoul, Korea) containing 10% fetal bovine serum (FBS; Thermo Fisher Scientific, Waltham, MA, USA) and 1% penicillin-streptomycin solution (P/S; Welgene). For viral infection, RD cells were exposed to HCoV-OC43 at a multiplicity of infection (MOI) of 0.01 (10⁶ PFU/mL), as described in earlier studies [14]. After inoculation with the virus-containing medium, cells were maintained in Minimum Essential Medium (MEM) supplemented with 2% FBS and 1% P/S.

For plaque assays, RD cells were seeded into 12-well plates and infected with the designated viral dilutions. Following infection, the cultures were overlaid with 0.3% agarose-containing medium and incubated for an additional four days. The resulting plaques were visualized by staining with a 0.25% crystal violet solution. Cell viability was assessed using an MTT assay as previously reported [14]. All procedures involving RD cells and HCoV-OC43 were performed under biosafety level 2 (BSL-2) conditions.

### Quantitative RT-PCR and Western blot analysis

To quantify coronavirus RNA levels in both cells and cell culture supernatants, quantitative RT-PCR was performed as described in a previous report [14]. In brief, total RNA was isolated from harvested cells and cell culture supernatants using PURE™ Total RNA Extraction Kit (Infusion Tech, Anyang, Korea), following the manufacturer’s guidelines. For the quantification of viral RNA in cell culture supernatants, total cellular RNA was added as carrier RNA. Complementary DNA (cDNA) was synthesized from equal amounts of total RNA using the M-MLV cDNA Synthesis Kit (Enzynomics, Daejeon, Republic of Korea). Quantitative real-time PCR (qRT-PCR) was subsequently carried out on a QuantStudio 3 Real-Time PCR System (Applied Biosystems, Thermo Fisher Scientific, Waltham, MA, USA) with 2 × Real-Time PCR Master Mix containing SYBR Green (BioFACT, Daejeon, Republic of Korea). RPL4 gene expression was used for normalization.

For detection of viral proteins, Western blotting was conducted as previously described [14]. HCoV-OC43–infected RD cells and cell culture supernatants were collected separately. Cells were lysed in lysis buffer (150 mM NaCl, 50 mM HEPES at pH 7.5, and 1% NP-40) supplemented with a protease inhibitor cocktail (Roche, Basel, Switzerland). Equal amounts of cell lysates (20 µg of total protein) and equal volumes of cell culture supernatants (20 µl) were subjected to SDS-PAGE and analyzed by Western blotting using an anti-HCoV-OC43 antibody. Mouse polyclonal anti-HCoV-OC43 antibodies were generated using the inactivated HCoV-OC43 particles in mice, as described previously [26]. Protein bands were visualized using the ChemiDoc™ Imaging System (Bio-Rad Laboratories, Hercules, CA, USA). HCoV-OC43 protein levels in cell lysates were normalized to GAPDH expression.

### Statistical analysis

Western blot and quantitative RT-PCR data were analyzed using one-way ANOVA followed by Dunnett’s multiple comparisons test in GraphPad Prism software, version 8.0.2 (GraphPad Software, Boston, MA, USA). A *p*-value of less than 0.05 was considered statistically significant. Dose–response curves were fitted using nonlinear regression analysis with a four-parameter logistic (4PL) model, and IC_50_ values were calculated from the fitted curves.

## Results

### *R. crispus* extract inhibits the expression of coronavirus protein

We investigated the antiviral activity of plant extracts and found that *R. crispus* extract has the potential to inhibit coronavirus replication. RD cells were infected with equal titers of coronavirus and subsequently treated with *R. crispus* extract. Viral replication was assessed by measuring coronavirus protein expression levels. Ethanol extract, water, n-hexane, and ethyl acetate (EA) fractions of *R. crispus* were evaluated for their inhibitory effects on HCoV-OC43 replication at 25 and 50 µg/ml. Among these, the n-hexane and EA fractions of *R. crispus* extract showed strong inhibitory effects on coronavirus protein expression (Figs 1A, 1B and S14). To further confirm these results, coronavirus-infected cells were treated with the n-hexane and EA fractions, and both fractions showed dose-dependent inhibitory effects on coronavirus protein expression in both cell lysates and cell culture supernatants (Figs 1C, 1D and S15). When the inhibitory effects of *R. crispus* were compared, stronger inhibition was observed in cell culture supernatants than in cell lysates. We also calculated the half-maximal inhibitory concentration (IC₅₀) of the n-hexane and EA fractions of *R. crispus*. The IC₅₀ of the EA fraction was lower than that of the n-hexane fraction in both cell lysates and media analyses. These results indicate that *R. crispus* extract (n-hexane and EA fractions) has the potential to inhibit coronavirus replication, with the EA fraction showing relatively stronger inhibitory activity than the n-hexane fraction.

**Fig 1:**
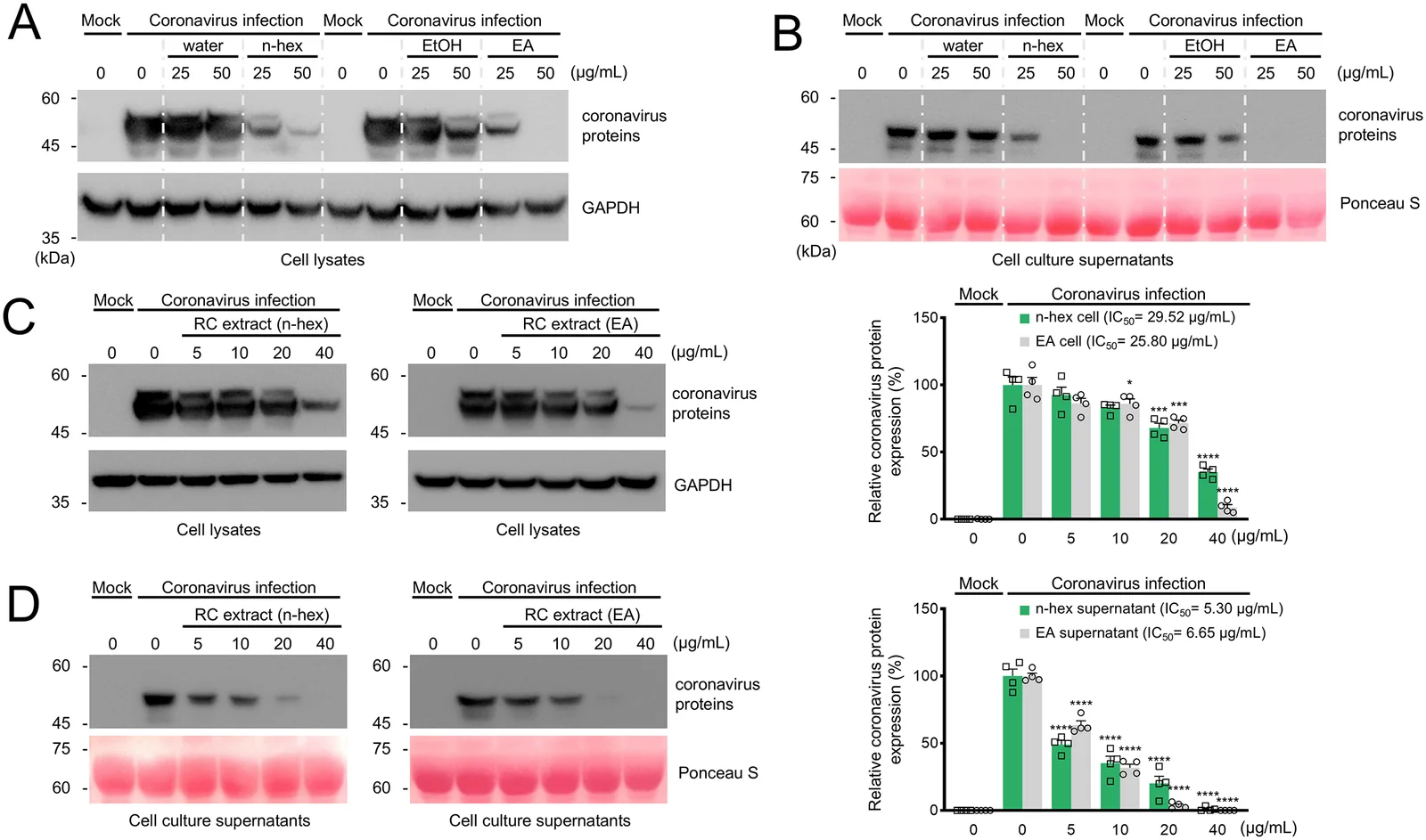
N-hexane and ethyl acetate fractions decrease HCoV-OC43 protein expression in a dose-dependent manner. **(A, B)** The *Rumex crispus* ethanol (EtOH) extract was partitioned into n-hexane (n-hex), ethyl acetate (EA), and water fractions. RD cells were infected with Human coronavirus (HCoV-OC43) and treated with the indicated concentrations of each fraction for 72 h. Coronavirus protein expression was detected using an anti-HCoV-OC43 antibody in cell lysates **(A)** and cell culture supernatants **(B)**. GAPDH was used as a loading control for cell lysates, and Ponceau S staining was used for cell culture supernatants. **(C)** The n-hexane and EA fractions of *R. crispus* were used to examine the inhibitory activity in cell lysates (top panel) and cell culture supernatants (bottom panel). Coronavirus-infected cells were treated with the indicated concentrations of R. crispus extract. **(D)** Relative human coronavirus protein expression levels were quantified and are shown in the bar graphs. The error bars represent the standard error of the mean (n = 4). Coronavirus-infected control vs. n-hex or EA treated groups, *: *p* < 0.05, **: *p* < 0.01, ***: *p* < 0.001, ****: *p* < 0.0001.

### *R. crispus* extract ameliorates coronavirus induced cytotoxicity

Coronavirus infection results in cytotoxicity, and antiviral molecules often reduce coronavirus-induced cytotoxicity. Because *R. crispus* extract decreased coronavirus protein expression, we examined whether *R. crispus* extract ameliorates coronavirus-induced cytotoxicity. First, we assessed the cytotoxicity of *R. crispus* extract by measuring cell viability. The MTT assay showed that *R. crispus* extracts (n-hexane and EA fractions) did not decrease cell viability up to 80 µg/mL (Fig 2A). Next, we examined whether *R. crispus* extract reduces coronavirus-induced cytotoxicity. While coronavirus infection decreased cell viability by up to 50%, treatment with *R. crispus* extract (n-hexane and EA fractions) significantly ameliorated coronavirus-induced cytotoxicity (Fig 2B). We also observed the protective effect of *R. crispus* extract by microscopy (Fig 2C). The number of cytotoxic cells was reduced following treatment with *R. crispus* extract. These results indicate that *R. crispus* ameliorates coronavirus-induced cytotoxicity.

**Fig 2:**
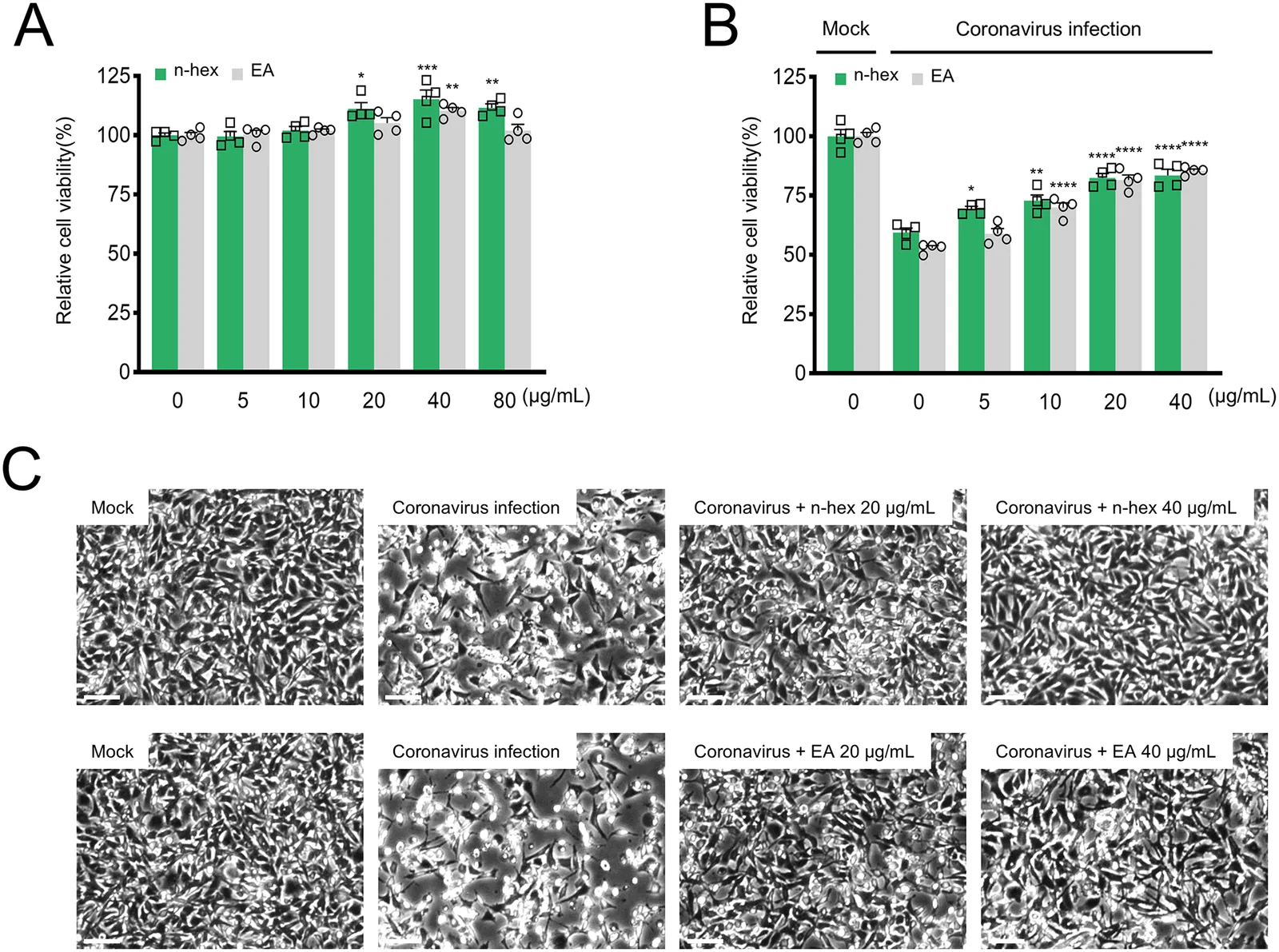
Treatment with n-hexane and EA fractions of *R. crispus* attenuates coronavirus-induced cytopathic effects. **(A)** RD cells were treated with the indicated concentrations of fractions for 24 h, and cell viability was assessed by an MTT assay. **(B)** RD cells were co-treated with human coronavirus and the indicated fractions for 72 h, followed by an MTT assay to determine cell viability. **(C)** Cell images were captured by light microscopy. RD cells were infected with HCoV-OC43 and treated with fractions for 72 h. Bar, 10 µm. The error bars represent the standard error of the mean (n = 4). Untreated control (A) or Coronavirus-infected control (B) vs. n-hex or EA treated groups, *: *p* < 0.05, **: *p* < 0.01, ***: *p* < 0.001, ****: *p* < 0.0001.

### *R. crispus* extract treatment inhibits the replication of coronavirus

Because *R. crispus* extract treatment decreased coronavirus protein expression and coronavirus-induced cytotoxicity, we further analyzed the antiviral effects of *R. crispus* extract against coronavirus. First, we examined the levels of coronavirus RNA in cells and cell culture supernatants to assess viral replication. We evaluated coronavirus RNA levels using the RNA-dependent RNA polymerase (RdRp) gene. Treatment with *R. crispus* extract decreased the RNA levels of human coronavirus in both cells and cell culture supernatants (Fig 3A). We also calculated the IC₅₀ values of *R. crispus* extract, and the IC₅₀ was lower in the cell culture supernatants than in the cell lysates, consistent with the Western blot results (Fig 3A). In addition, the IC₅₀ of the EA fraction was lower than that of the n-hexane fraction.

**Fig 3:**
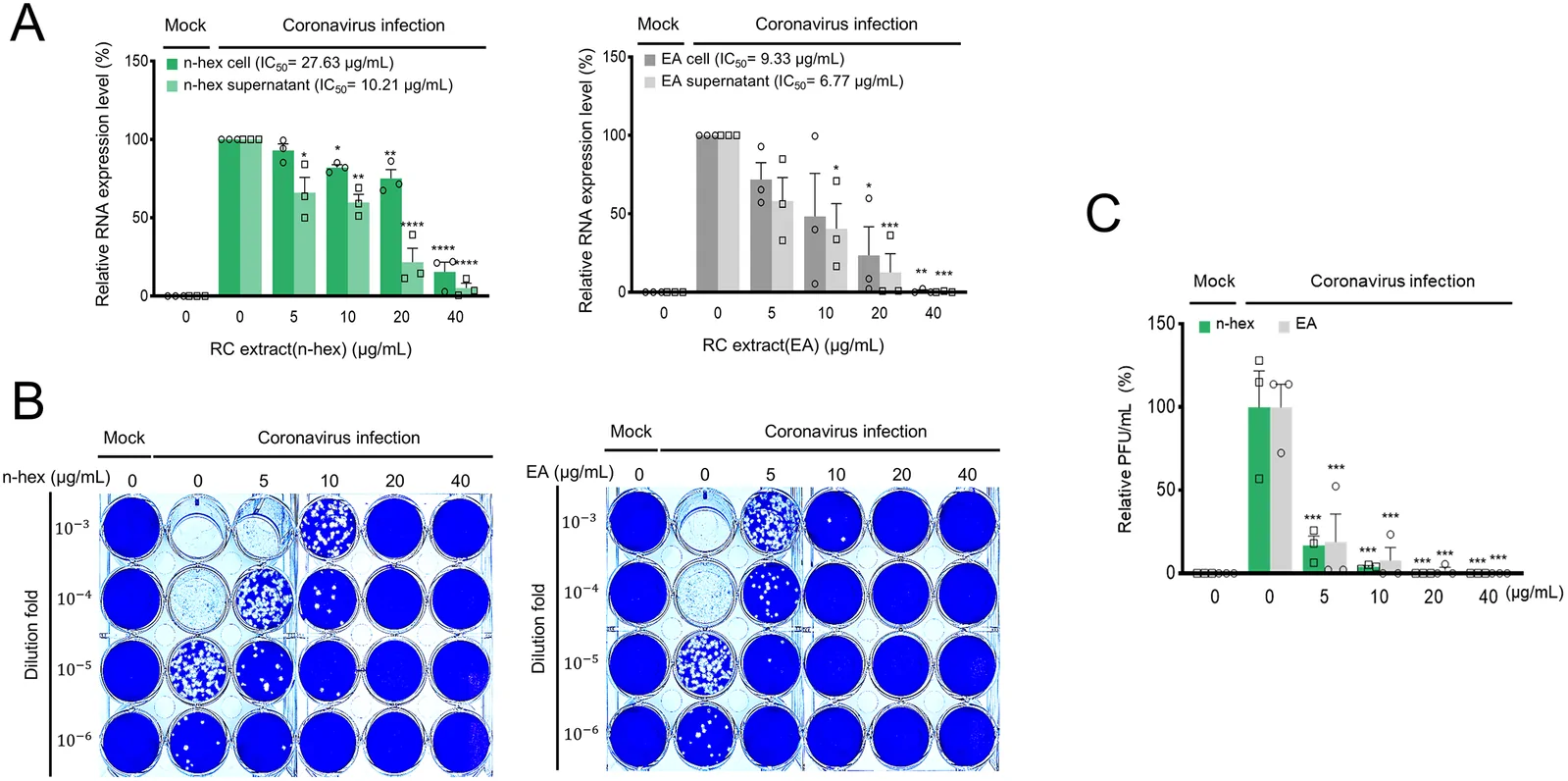
*R. crispus* extract treatment inhibits the production of coronavirus particles. **(A)** *R. crispus* extract treatment inhibits the level of human coronavirus RNA in cells. Coronavirus-infected cells were treated with the n-hex fraction (left) and EA fraction (right). The level of coronavirus RNA was quantified by qRT-PCR using the RNA dependent RNA polymerase (RdRp) gene. **(B)** *R. crispus* extract treatment reduces the formation of coronavirus-induced plaques. The cell culture supernatants were collected and examined by a plaque formation assay. **(C)** The number of plaques was quantified and is shown in the graph. Coronavirus-infected control vs. n-hex or EA treated groups, *: *p* < 0.05, **: *p* < 0.01, ***: *p* < 0.001, ****: *p* < 0.0001.

Because coronavirus RNA in the cell culture supernatants is mainly derived from viral particles, these results indicate that *R. crispus* extract inhibits coronavirus replication. Next, we examined the production of coronavirus particles following treatment with *R. crispus* extract. The cell culture supernatants from coronavirus-infected cells treated with *R. crispus* extract were collected and used for a plaque formation assay. Various dilutions of the cell culture supernatants were used to evaluate the number of plaques, and *R. crispus* extract treatment resulted in a dramatic decrease in plaque formation, indicating that *R. crispus* extract reduced the formation of coronavirus-induced plaques (Figs 3B and 3C). These results collectively indicate that *R. crispus* treatment inhibits coronavirus replication.

Finally, we directly observed the effect of *R. crispus* extract on coronavirus production by scanning electron microscopy. Infection with human coronavirus resulted in the formation of numerous aggregated viral particles on the surfaces of infected cells, and higher magnification revealed individual coronavirus particles (Figs 4A and 4B). Treatment with *R. crispus* extract (n-hexane and EA fractions) reduced the number of coronavirus particles on the cell surface (Fig 4A and 4B). Viral particles were barely observed following treatment with *R. crispus* extract at 40 µg/ml. These results indicate that *R. crispus* extract treatment decreases the production of coronavirus on the surfaces of infected cells.

**Fig 4:**
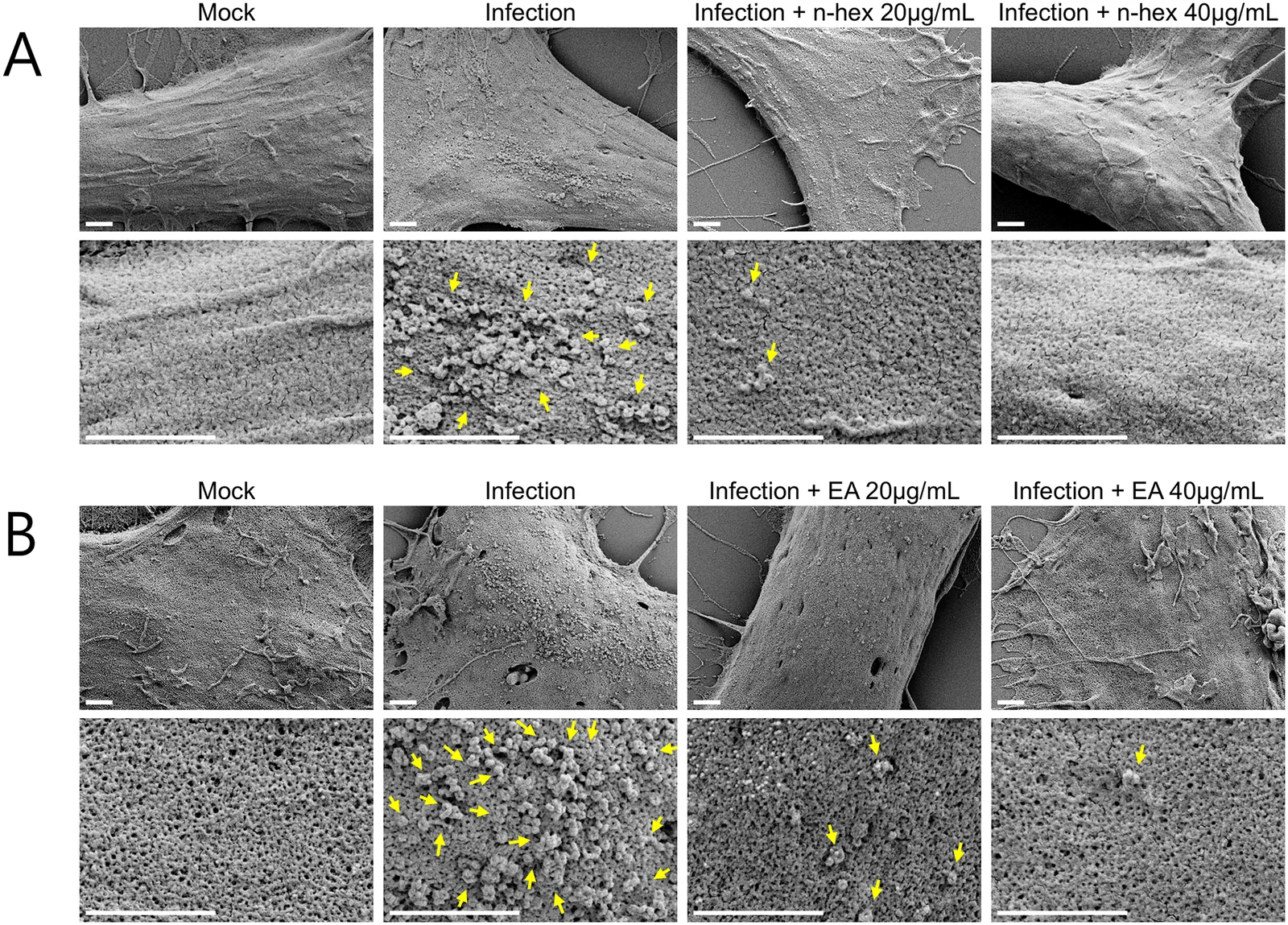
Treatment with n-hexane and EA fractions suppresses coronavirus release, as observed by scanning electron microscopy (SEM). RD cells were co-treated with human coronavirus and n-hexane **(A)** or ethyl acetate **(B)** fractions for 72 h. Cells were processed for SEM analysis. Viral particles are indicated by arrows. Dose-dependent inhibition of viral release was observed. Scale bar, 1 μm.

### Isolation of single compounds from *R. crispus* extract

To identify potential inhibitors from the roots of *R. crispus,* we analyzed the ethanol extract, n-hexane fraction, EA fraction, and water fraction using HPLC (Fig 5A). While the ethanol extract and water fraction showed a small number of peaks, the n-hexane and EA fractions exhibited many prominent peaks (Fig 5A). Therefore, five and eight compounds were isolated from the n-hexane and ethyl acetate fractions, respectively, using silica gel and C-18 column chromatography. Their structures were elucidated by comparing NMR signals with those previously reported (Figs S1–S13). The compounds were identified as epicatechin (**1**) [27], epicatechin-O-gallate (**2**) [28], 6-hydroxymusizin-8-O-β-glucopyranoside (**3**) [29], nepodin-8-O-β-glucopyranoside (**4**) [30], torachrysone-8-O-β-glucopyranoside (**5**) [27], emodin-8-O-β-glucopyranoside (**6**) [31], chrysophanol-8-O-β-glucopyranoside (**7**) [32], nepodin-8-O-β-glucopyranoside (**8**) [33], citreorosein (**9**) [27], 6′-acetyl-chrysophanol-8-O-β-glucopyranoside (**10**) [27], nepodin (**11**) [32], emodin (**12**) [32], and chrysophanol (**13**) [32] (Fig 5B).

**Fig 5:**
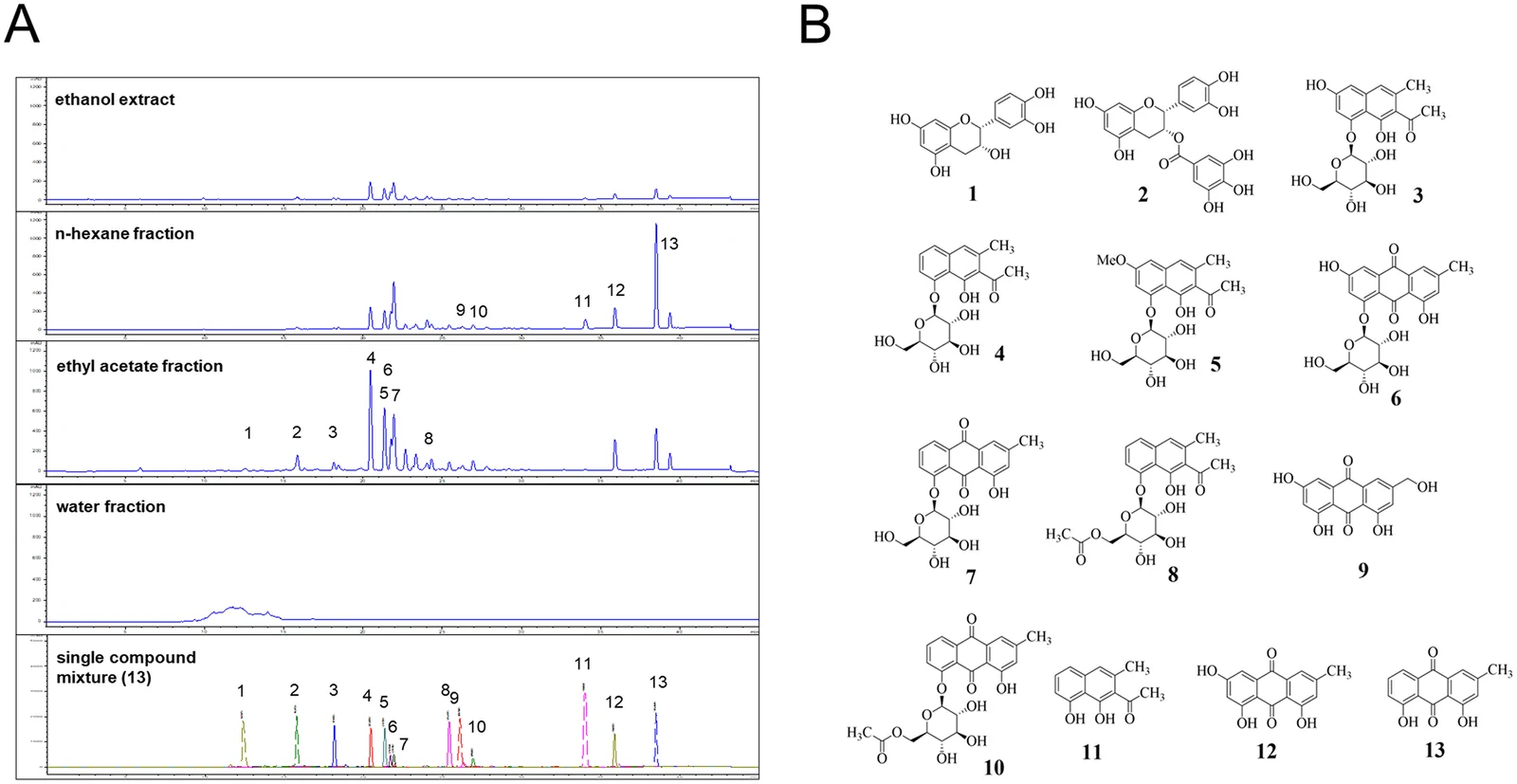
HPLC analysis of *R. crispus* extract. **(A)** HPLC chromatograms of ethanol extract, n-hexane, ethyl acetate, and water fractions, and the isolated compound mixture at 254 nm. **(B)** Chemical structure of single compounds. epicatechin (**1**), epicatechin-*O*-gallate (**2**), 6-hydroxymusizin-8-*O*-*β*-glucopyranoside (**3**), nepodin-8-*O*-*β*-glucopyranoside (**4**), torachrysone-8-*O*-*β*-glucopyranoside (**5**), emodin-8-*O*-*β*-glucopyranoside (**6**), chrysophanol-8-*O*-*β*-glucopyranoside (**7**), nepodin-8-*O*-*β*-glucopyranoside (**8**), citreorosein (**9**), 6′-acetyl-chrysophanol-8-*O*-*β*-glucopyranoside (**10**), nepodin (**11**), emodin (**12**) and chrysophanol (**13**).

Because thirteen compounds were isolated from *R. crispus* extract, we investigated which compound could inhibit coronavirus replication. RD cells were infected with human coronavirus OC43 (HCoV-OC43) and treated with each compound at concentrations of 10 µM and 20 µM, respectively. Among these, emodin (**12**) was identified as a potent inhibitor of viral protein expression (Figs 6 and S16). In addition, citreorosein (**9**), a structural analogue of emodin (**12**), also showed inhibitory activity against coronavirus replication. However, the abundance of citreorosein in *R. crispus* extract is very low (Fig 5A), and its inhibitory activity is weaker than that of emodin.

**Fig 6:**
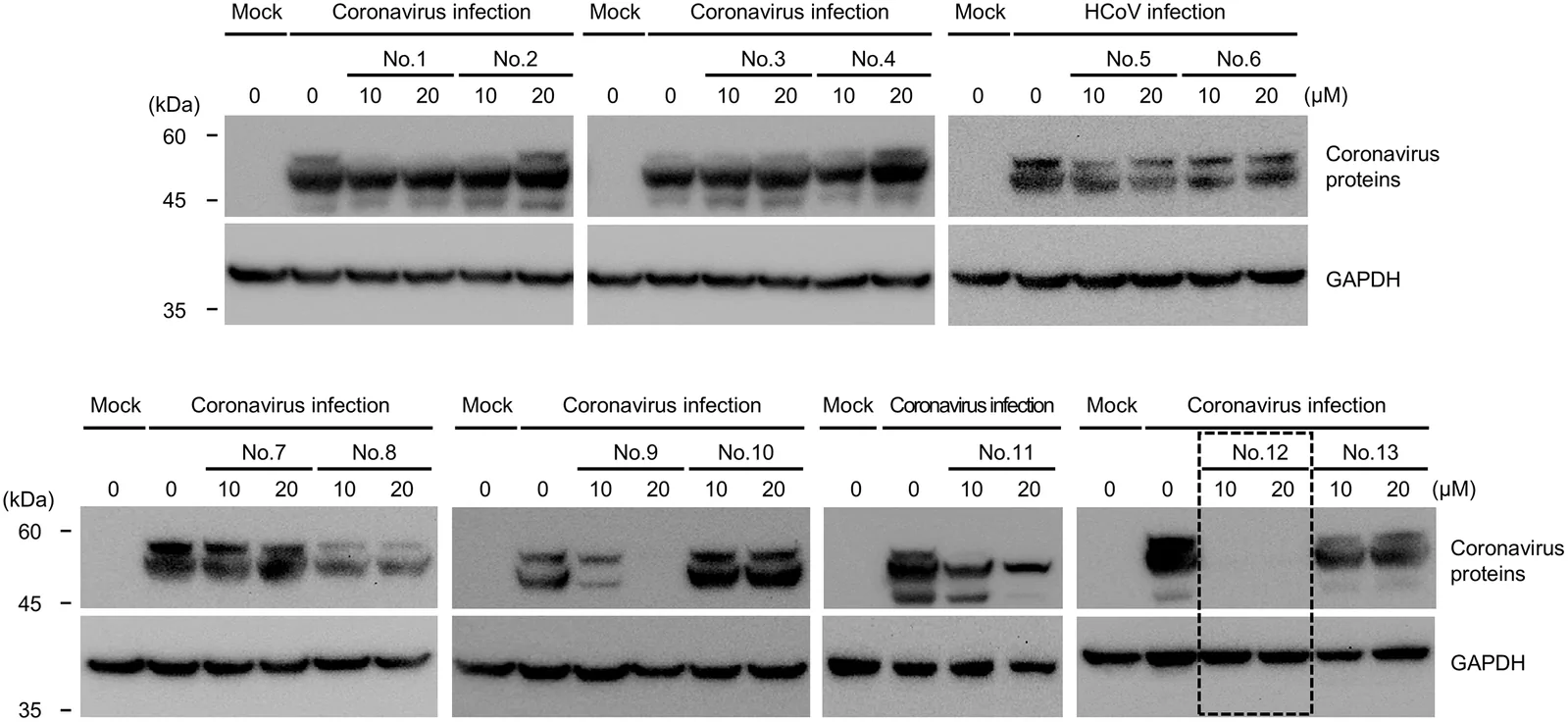
Screening of antiviral activity of the isolated compounds by Western blot analysis. RD cells were infected with human coronavirus and treated with individual compounds for 72 h. Cell lysates were collected, and coronavirus protein expression was analyzed by Western blot. Emodin (**12**) showed the highest antiviral activity.

These results suggest that emodin may be one of the active compounds contributing to the inhibitory effects of *R. crispus* extract on coronavirus replication. Previous studies have demonstrated that emodin is a potential inhibitor of coronavirus [25]; however, the reduction of coronavirus protein expression by emodin treatment has not been fully evaluated. To compare the antiviral activity of emodin, we treated coronavirus-infected cells with emodin under the same conditions used for *R. crispus* extract experiments and examined antiviral activity using Western blot analysis (Figs 7A, 7B and S17). Emodin inhibited coronavirus protein expression in a dose-dependent manner in both cell lysates and cell culture supernatants (Fig 7A). Moreover, the IC₅₀ values of emodin were 1.655 µM in the cell culture supernatants and 2.500 µM in the cell lysates, respectively (Fig 7B). These IC₅₀ values are substantially lower than those of *R. crispus* extract. In addition, we examined the effect of emodin on cell viability and found that treatment with emodin at concentrations up to 20 µM did not significantly affect cell viability (Fig 7C).

**Fig 7:**
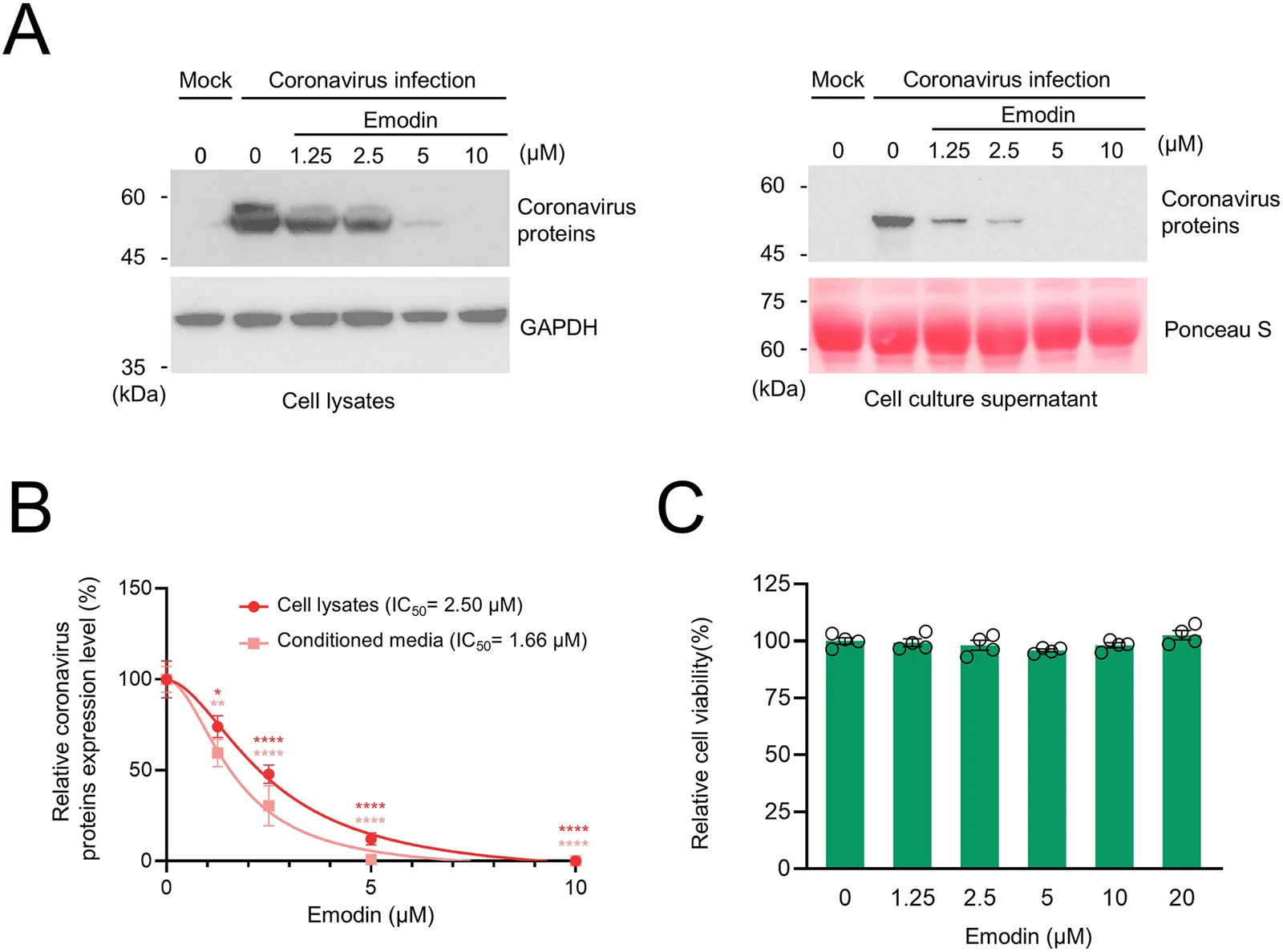
Emodin reduces viral protein expression in RD cells. **(A)** RD cells were infected with human coronavirus and treated with the indicated concentrations of emodin for 72 h. The levels of coronavirus proteins were examined by Western blot in cell lysates (left panel) and cell culture supernatants (right panel). **(B)** Dose–response curves show the relative coronavirus protein expression levels normalized to GAPDH or total protein. Error bars represent the standard error of the mean (n = 4). Coronavirus-infected control vs emodin treated groups, *: *p* < 0.05, **: *p* < 0.01, ***: *p* < 0.001, ****: *p* < 0.0001. **(C)** RD cells were treated with the indicated concentrations of emodin for 24 h, and cell viability was assessed by an MTT assay.

## Discussion

Anthraquinones (emodin, rhein, and aloe-emodin) were reported as candidates in the treatment of SARS-CoV-2 [34]. It was reported that emodin can block the open-reading-frame 3a ion channel of SARS-CoV and HCoV-OC43 [25]. The roots of *R. crispus* contain a variety of anthraquinone derivatives, such as emodin, chrysophanol, physcion and emodin-8-O-*β*-glucopyranoside [35]. Emodin was reported to have therapeutic potential against cancer, diabetes, and chronic inflammatory diseases [36].

In this study, 13 single compounds were isolated from this plant to find potential inhibitors that suppress the proliferation of human coronavirus. As a result, two epicatechin derivatives (**1**, **2**), five naphthalene derivatives (**3**, **4**, **5**, **8**, **11**), and six anthraquinone derivatives (**6**, **7**, **9**, **10**, **12**, **13**) were isolated from the roots of *R. crispus* and identified (Fig 5). In addition, it was found that six compounds (**4–7**, **12**, **13**) were the major peaks in the ethanol extract. These compounds (**1**–**13**) tended to have different effects on inhibiting viral proliferation in cells and cell culture supernatants, depending on their scaffold. After extensive screening, we demonstrated that emodin, a major anthraquinone, inhibits coronavirus replication. It was also found that the amount of emodin (**12**), antiviral inhibitor, was present in the ethyl acetate fraction and *n*-hexane fraction of *R. crispus*. The amount of emodin in the water fraction was negligible or very low. Similarly, the antiviral activities of ethyl acetate and n-hexane fractions are comparable, while the water fraction was low. We also demonstrated that emodin exhibits similar antiviral activity, including a reduction in virus-induced cytopathic effects and the reduction of coronavirus RNA and protein. These results indicate that the ethyl acetate and n-hexane fractions of *R. crispus* exhibit antiviral activity, to which emodin may contribute, along with other potentially bioactive constituents.

Although vaccines and antiviral agents against coronaviruses have been developed, alternative therapeutic strategies remain desirable. In this study, we demonstrated that root extracts of *R. crispus* exhibit antiviral activity against human coronavirus, to which the bioactive anthraquinone emodin may contribute, along with other bioactive constituents. Based on these findings, emodin may represent a promising lead compound for the development of novel antiviral agents against coronaviruses. Furthermore, structural modification of emodin may yield more potent derivatives, and future studies, including in vivo animal experiments, will be essential to evaluate their therapeutic efficacy.

Although the antiviral activity of emodin against coronaviruses has been previously reported, the novelty of the present study lies in demonstrating the antiviral activity of *R. crispus* root fractions against HCoV-OC43 and identifying emodin as one of the bioactive constituents associated with this activity. Previous studies have identified emodin and other anthraquinones in *R. crispus* roots; however, their association with the anti-coronavirus activity of these fractions has not been fully investigated. In this study, we demonstrated that the n-hexane and ethyl acetate fractions effectively inhibited HCoV-OC43 replication and confirmed the presence of emodin in the active fractions through phytochemical analysis. Furthermore, purified emodin independently exhibited antiviral activity against HCoV-OC43. These findings suggest that emodin may contribute to the antiviral effects of *R. crispus* root fractions. Nevertheless, other bioactive constituents and potential synergistic interactions may also contribute to the observed activity. Further studies are needed to determine the relative contributions of these compounds and their possible synergistic effects. Taken together, our findings provide new evidence for the antiviral potential of *R. crispus* root fractions and establish a foundation for further investigation of plant-derived antiviral agents against coronaviruses.

## Supporting information

S1 Fig 1H-NMR signal of epicatechin (1) (1H NMR: 300MHz, MeOD-d4). (PDF)

S2 Fig 1H- and 13C-NMRsignals of epicatechin-O-gallate (2) (1H NMR: 300 MHz,13C NMR:75 MHz, MeOD-d4). (PDF)

S3 Fig 1H- and 13C-NMRsignals of 6-hydroxymusizin-8-O-β-glucopyranoside (3) (1H NMR: 300 MHz,13C NMR:75 MHz, MeOD-d4). (PDF)

S4 Fig 1H- and 13C-NMRsignals of nepodin-8-O-β-glucopyranoside (4) (1H NMR: 300 MHz,13C NMR:75 MHz, MeOD-d4). (PDF)

S5 Fig 1H- and 13C-NMRsignals of torachrysone-8-O-β-glucopyranoside (5) (1H NMR: 300 MHz,13C NMR:75 MHz, MeOD-d4). (PDF)

S6 Fig 1H- and 13C-NMRsignals of emodin-8-O-β-glucopyranoside (6) (1H NMR: 300 MHz,13C NMR:75 MHz, DMSO-d6). (PDF)

S7 Fig 1H- and 13C-NMRsignals of chrysophanol-8-O-β-glucopyranoside (7) (1H NMR: 300 MHz,13C NMR:75 MHz, DMSO-d6). (PDF)

S8 Fig 1H- and 13C-NMRsignals of nepodin-8-O-β-glucopyranoside (8) (1H NMR: 300 MHz,13C NMR:75 MHz, MeOD-d4). (PDF)

S9 Fig 1H- and 13C-NMRsignals of citreorosein (9) (1H NMR: 300 MHz,13C NMR:75 MHz, DMSO-d6). (PDF)

S10 Fig 1H- and 13C-NMRsignals of 6′-acetyl-chrysophanol-8-O-β-glucopyranoside (10) (1H NMR: 300 MHz,13C NMR:75 MHz, DMSO-d6). (PDF)

S11 Fig 1H- and 13C-NMRsignals of nepodin (11) (1H NMR: 300 MHz,13C NMR:75 MHz, MeOD-d4). (PDF)

S12 Fig 1H- and 13C-NMRsignals of emodin (12) (1H NMR: 300 MHz,DMSO-d6). (PDF)

S13 Fig 1H-signal of chrysophanol (13) (1H NMR: 300 MHz, DMSO-d6). (PDF)

S14 Fig Uncropped western blot images corresponding to Fig 1A and 1B. (PDF)

S15 Fig Uncropped western blot images corresponding to Fig 1C and 1D. (PDF)

S16 Fig Uncropped western blot images corresponding to Fig 6. (PDF)

S17 Fig Uncropped western blot images corresponding to Fig 7. (PDF)
