# Supplementary material for: Antiviral activity of Rumex crispus extract and its bioactive constituent emodin against human coronavirus OC43
Source: PLoS One. 2026 Nov 5;21(11):e0362181. doi: 10.1371/journal.pone.0362181 (PMC13739101; doi:10.1371/journal.pone.0362181)
Supplement: Supplementary file 1 [file pone.0362181.s001.pdf]

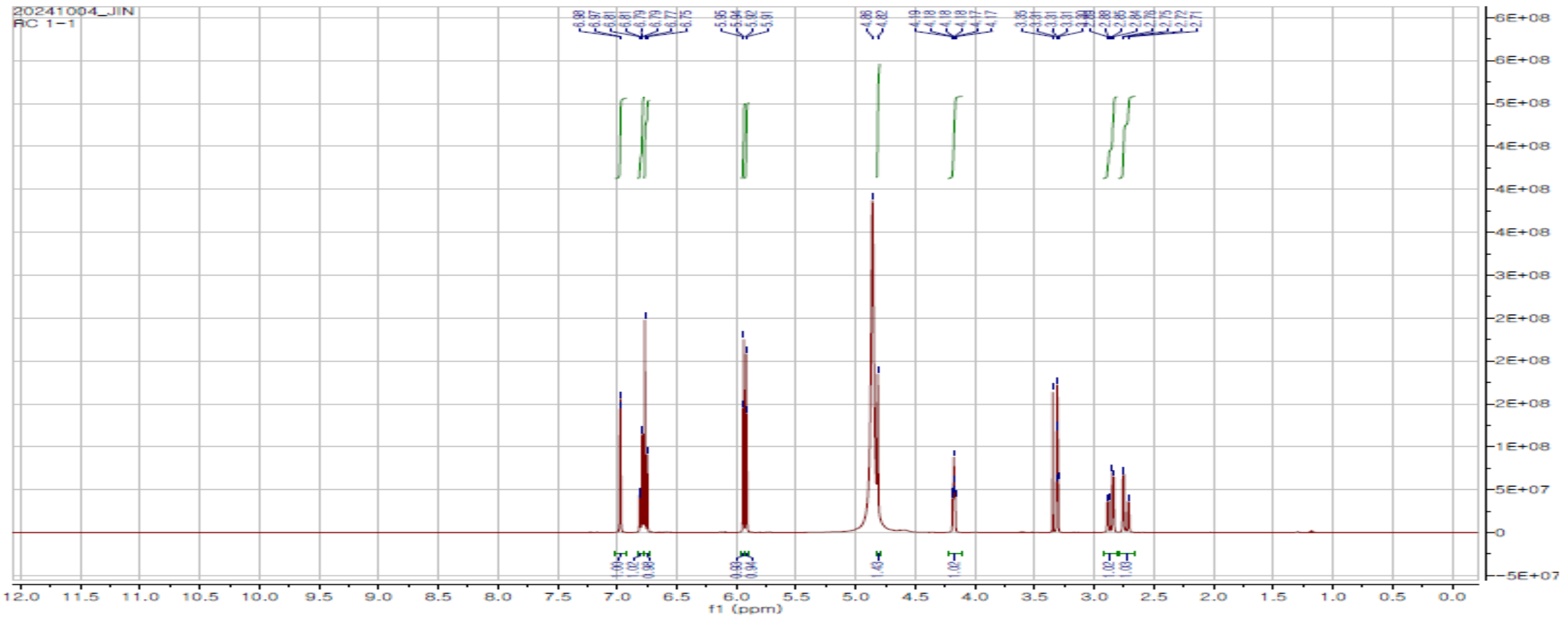

S1 Fig. ^{1}H-NMR signal of epicatechin (1) (^{1}H NMR: 300MHz, MeOD-d_4).
