# Supplementary material for: Antiviral activity of Rumex crispus extract and its bioactive constituent emodin against human coronavirus OC43
Source: PLoS One. 2026 Nov 5;21(11):e0362181. doi: 10.1371/journal.pone.0362181 (PMC13739101; doi:10.1371/journal.pone.0362181)
Supplement: Supplementary file 3 [file pone.0362181.s003.pdf]

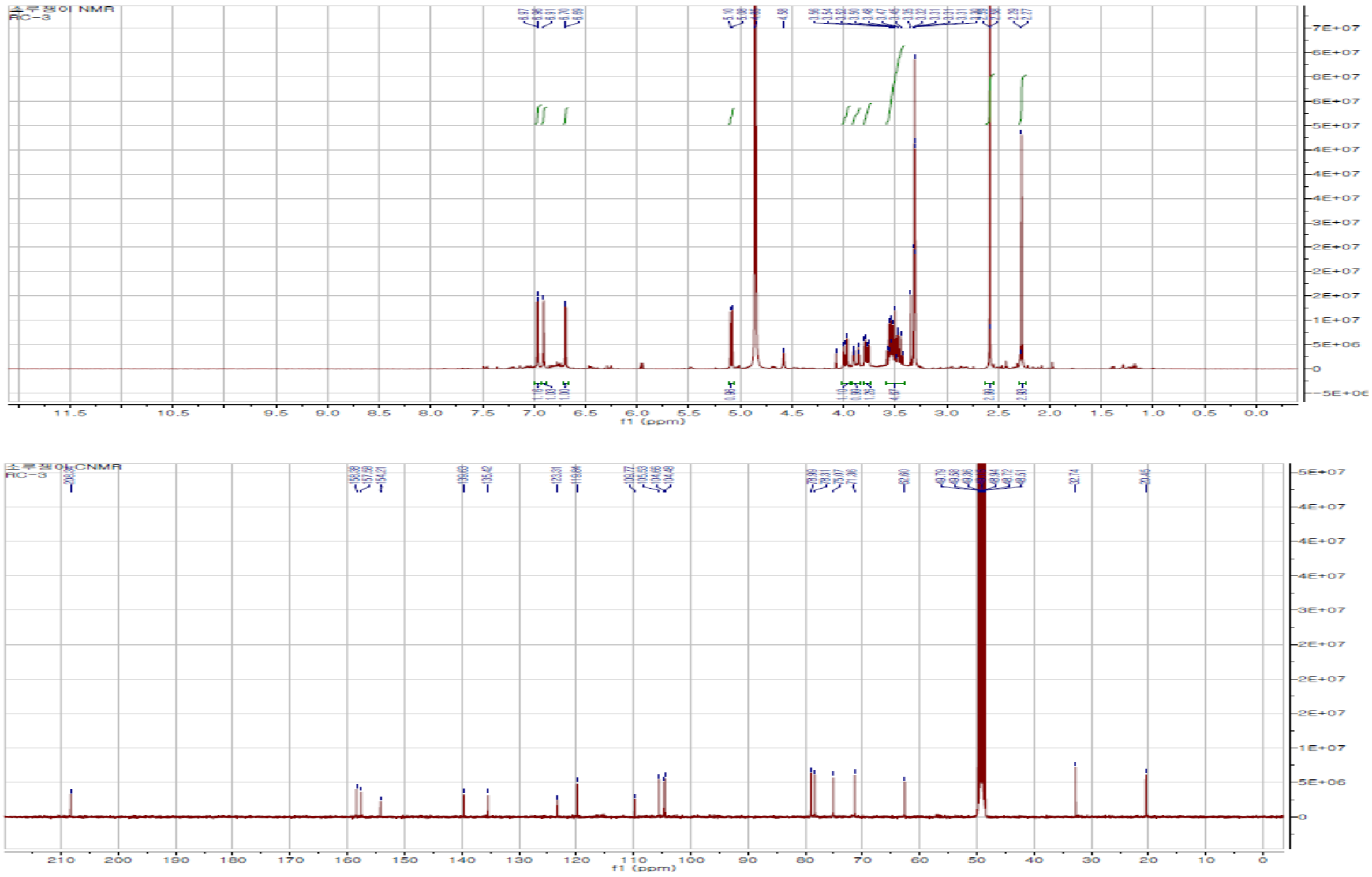

**S3 Fig.** $^{1}$H- and $^{13}$C-NMRsignals of 6-hydroxymusizin-8-O-β-glucopyranoside (3) ($^{1}$H NMR: 300 MHz,$^{13}$C NMR:75 MHz, MeOD-$d_4$).
