# Supplementary material for: Antiviral activity of Rumex crispus extract and its bioactive constituent emodin against human coronavirus OC43
Source: PLoS One. 2026 Nov 5;21(11):e0362181. doi: 10.1371/journal.pone.0362181 (PMC13739101; doi:10.1371/journal.pone.0362181)
Supplement: Supplementary file 10 [file pone.0362181.s010.pdf]

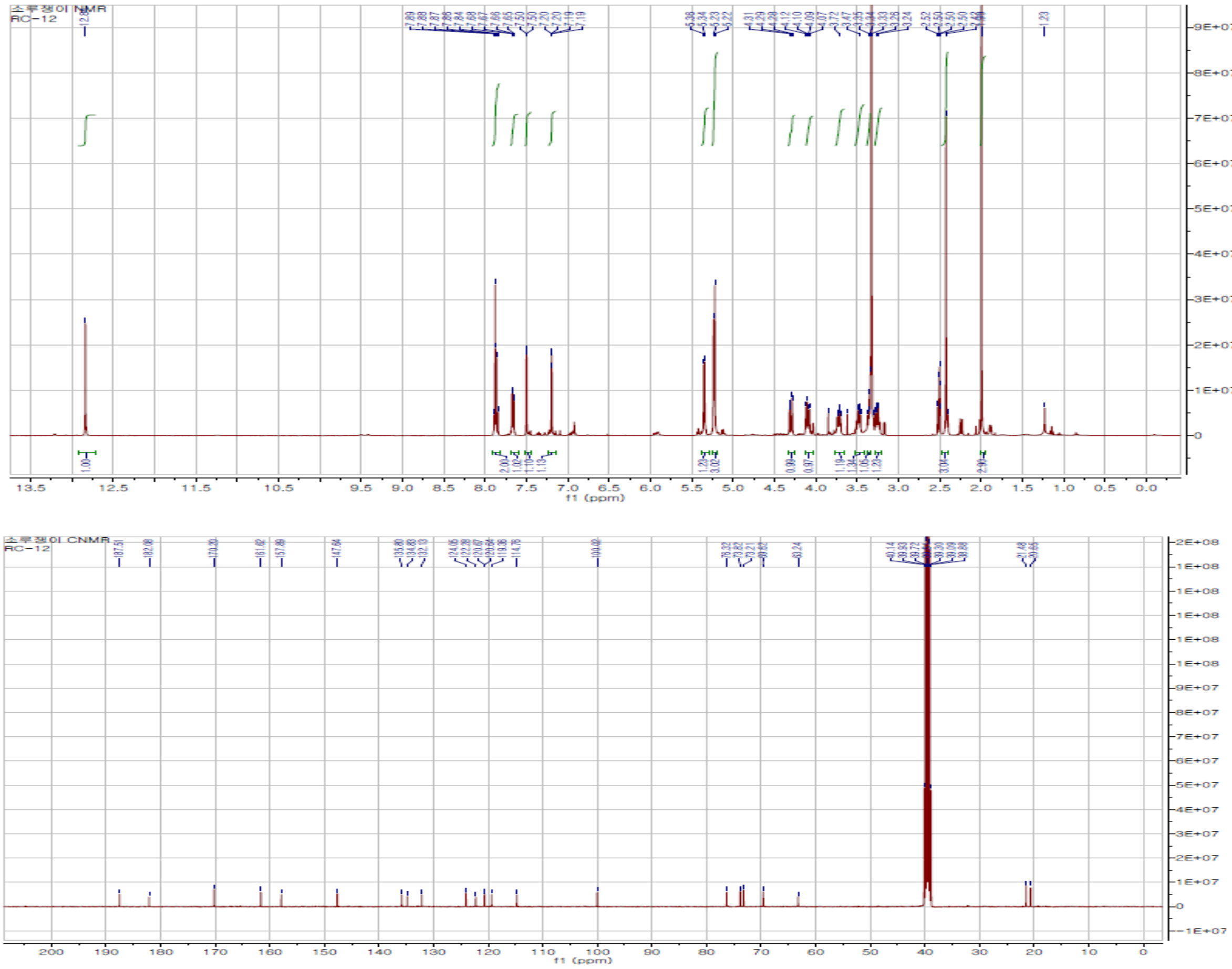

S10 Fig. ^{1}H- and ^{13}C-NMRsignals of 6′-acetyl-chrysophanol-8-O-β-glucopyranoside (10) (^{1}H NMR: 300 MHz,^{13}C NMR:75 MHz, DMSO-d_6).
