# Supplementary material for: Antiviral activity of Rumex crispus extract and its bioactive constituent emodin against human coronavirus OC43
Source: PLoS One. 2026 Nov 5;21(11):e0362181. doi: 10.1371/journal.pone.0362181 (PMC13739101; doi:10.1371/journal.pone.0362181)
Supplement: Supplementary file 18 [file pone.0362181.s018.docx]

Reviewer's Responses to Questions

Title: Rumex crispus Extract Exhibits Antiviral Activity Against Coronavirus and Contains the Active Compound Emodin

(Cho et al., PONE-D-26-16257)

We appreciate the reviewer’s constructive feedback. We have made changes to the manuscript and have addressed all comments in detail below.

Response to the Editor and Journal Requirements

1. Please ensure that your manuscript meets PLOS ONE's style requirements, including those for file naming.

🡪 We have revised the manuscript to address PLOS ONE’s style requirements. Specifically, we removed postal codes from the author affiliations and omitted the affiliation from the correspondence section, which now reads:

*Correspondence:

E-mail: junsoo@yonsei.ac.kr (JP)

We also adjusted the font sizes and bold formatting of the level 1 and level 2 headings, standardized the formatting of figure numbers, and added a Supporting information section. In addition, we placed the figure legends within the Results section, immediately after the paragraphs in which the respective figures are first cited. We have also checked the submission file names against the journal’s requirements.

2. We note that this submission includes NMR spectroscopy data. We would recommend that you include the following information in your methods section or as Supporting Information files:

1) The make/source of the NMR instrument used in your study, as well as the magnetic field strength. For each individual experiment, please also list: the nucleus being measured; the sample concentration; the solvent in which the sample is dissolved and if solvent signal suppression was used; the reference standard and the temperature.
2) A list of the chemical shifts for all compounds characterised by NMR spectroscopy, specifying, where relevant: the chemical shift (δ), the multiplicity and the coupling constants (in Hz), for the appropriate nuclei used for assignment.
3) The full integrated NMR spectrum, clearly labelled with the compound name and chemical structure.

We also strongly encourage authors to provide primary NMR data files, in particular for new compounds which have not been characterised in the existing literature. Authors should provide the acquisition data, FID files and processing parameters for each experiment, clearly labelled with the compound name and identifier, as well as a structure file for each provided dataset

🡪Thank you for these recommendations. We have revised the Methods section and Supporting Information to clarify the NMR measurements and compound identification.

We added NMR data figures files in Supporting information (S1 Fig- S13 Fig).

All 13 isolated compounds are known compounds and were identified by comparison of their NMR data with the published data cited in the manuscript. No new compounds are reported in this study.

3. Thank you for stating the following in the Acknowledgments Section of your manuscript:

"This study was supported by the Cooperative Research Program (RS-2022-RD010239) of the Rural Development Administration, Republic of Korea."

We note that you have provided additional information within the Acknowledgements Section that is not currently declared in your Funding Statement. Please note that funding information should not appear in the Acknowledgments section or other areas of your manuscript. We will only publish funding information present in the Funding Statement section of the online submission form.

Please remove any funding-related text from the manuscript and let us know how you would like to update your Funding Statement. Currently, your Funding Statement reads as follows:

"This study was supported by the Cooperative Research Program (RS-2022-RD010239) of the Rural Development Administration (https://www.rda.go.kr), Republic of Korea, awarded to J.P.. The funders had no role in study design, data collection and analysis, decision to publish, or preparation of the manuscript."

🡪We have removed the funding-related text from the Acknowledgments section. We confirm that the current Funding Statement is complete and accurate, apart from a duplicated period after “J.P.” We request that the statement be retained with this punctuation corrected, as follows:

“This study was supported by the Cooperative Research Program (RS-2022-RD010239) of the Rural Development Administration (https://www.rda.go.kr), Republic of Korea, awarded to J.P. The funders had no role in study design, data collection and analysis, decision to publish, or preparation of the manuscript.”

4. PLOS ONE now requires that authors provide the original uncropped and unadjusted images underlying all blot or gel results reported in a submission’s figures or Supporting Information files. This policy and the journal’s other requirements for blot/gel reporting and figure preparation are described in detail at https://journals.plos.org/plosone/s/figures#loc-blot-and-gel-reporting-requirements and https://journals.plos.org/plosone/s/figures#loc-preparing-figures-from-image-files. When you submit your revised manuscript, please ensure that your figures adhere fully to these guidelines and provide the original underlying images for all blot or gel data reported in your submission. See the following link for instructions on providing the original image data: https://journals.plos.org/plosone/s/figures#loc-original-images-for-blots-and-gels.

🡪 We have provided the original, uncropped and unadjusted Western blot images underlying Fig 1A, Fig 1B, Fig 6, and Fig 7 in a single PDF file, S1_raw_images.pdf, as Supporting Information. Each image is labelled with the corresponding figure and panel number.

5. Please include captions for your Supporting Information files at the end of your manuscript, and update any in-text citations to match accordingly.

🡪We have added captions for all Supporting Information files in the “Supporting information” section at the end of the manuscript. The corresponding in-text citations have also been checked and revised to match the Supporting Information labels.

6. PLOS ONE now requires that authors provide the original uncropped and unadjusted images underlying all blot or gel results reported in a submission’s figures or Supporting Information files. This policy and the journal’s other requirements for blot/gel reporting and figure preparation are described in detail at https://journals.plos.org/plosone/s/figures#loc-blot-and-gel-reporting-requirements and https://journals.plos.org/plosone/s/figures#loc-preparing-figures-from-image-files. When you submit your revised manuscript, please ensure that your figures adhere fully to these guidelines and provide the original underlying images for all blot or gel data reported in your submission. See the following link for instructions on providing the original image data: https://journals.plos.org/plosone/s/figures#loc-original-images-for-blots-and-gels.

🡪As noted in our response to item 4, the original Western blot images are provided in S1_raw_images.pdf as Supporting Information.

7. If the reviewer comments include a recommendation to cite specific previously published works, please review and evaluate these publications to determine whether they are relevant and should be cited. There is no requirement to cite these works unless the editor has indicated otherwise.

🡪We have reviewed the publications and patent cited by Reviewer #1 and considered their relevance to the manuscript. The Introduction and Discussion have been revised to acknowledge the previously reported anti-coronavirus activity of emodin and to clarify the contribution of the present study.

Reviewer’s comments

Reviewer #1:

This study revealed that Rumex crispus extract possesses antiviral properties against coronavirus, primarily attributed to the active compound emodin. The primary strength of this study lies in its logical workflow from natural products to bioactivity, which identified emodin as the probable antiviral agent in Rumex crispus root extracts. Nonetheless, the novelty is somewhat limited as the anti-coronavirus effects of emodin have already been established.
<https://pubmed.ncbi.nlm.nih.gov/16730806/>
<https://pmc.ncbi.nlm.nih.gov/articles/PMC7114100/>
<https://patents.google.com/patent/CN103156830A/en>
<https://pmc.ncbi.nlm.nih.gov/articles/PMC9433657/>

The unique contribution of this study is not in identifying the ability of emodin to inhibit coronaviruses, but in associating this effect with R. crispus root fractions and conducting a phytochemical analysis that locates emodin in the active fractions. To enhance the manuscript, it should clearly articulate what is novel beyond the known fact that "emodin was previously known." For example, it should specify whether R. crispus is a previously unreported source, whether the fractionation process significantly increases emodin concentration, or whether the plant produces a consistent extract with improved practical properties compared to purified emodin.

-> Thank you for your insightful comment. We agree that the antiviral activity of emodin against coronaviruses has been previously reported and that the novelty of our study should be more clearly emphasized. The primary contribution of our study is the demonstration of (1) the antiviral activity of *Rumex crispus* root fractions against HCoV-OC43 and (2) the identification of emodin as one of the bioactive constituents associated with this activity.

In response to the reviewer's suggestion, we have revised the Title, Introduction and Discussion to clarify the novelty of our findings. We have also acknowledged that further quantitative analysis and comparative studies are needed to determine whether fractionation enriches emodin and whether other constituents contribute to the observed antiviral activity.

- Title: Antiviral Activity of Rumex crispus Extract and Its Bioactive Constituent Emodin Against Human Coronavirus OC43

- Introduction: Although emodin has previously been reported to exhibit antiviral activity against coronaviruses, the antiviral potential of Rumex crispus root extracts and their fractions remains poorly understood. In this study, we evaluated the antiviral activity of *R. crispus* root extracts and solvent fractions against HCoV-OC43. Furthermore, we performed phytochemical analysis to identify bioactive constituents associated with the observed antiviral activity. Our findings demonstrate the anti-coronavirus activity of *R. crispus* root fractions and identify emodin as one of the compounds potentially contributing to this effect.

- Discussion: Although the antiviral activity of emodin against coronaviruses has been previously reported, the novelty of the present study lies in demonstrating the antiviral activity of R. crispus root fractions against HCoV-OC43 and identifying emodin as one of the bioactive constituents associated with this activity. Previous studies have identified emodin and other anthraquinones in R. crispus roots; however, their association with the anti-coronavirus activity of these fractions has not been fully investigated. In this study, we demonstrated that the n-hexane and ethyl acetate fractions effectively inhibited HCoV-OC43 replication and confirmed the presence of emodin in the active fractions through phytochemical analysis. Furthermore, purified emodin independently exhibited antiviral activity against HCoV-OC43. These findings suggest that emodin may contribute to the antiviral effects of R. crispus root fractions. Nevertheless, other bioactive constituents and potential synergistic interactions may also contribute to the observed activity. Further studies are needed to determine the relative contributions of these compounds and their possible synergistic effects. Taken together, our findings provide new evidence for the antiviral potential of R. crispus root fractions and establish a foundation for further investigation of plant-derived antiviral agents against coronaviruses.

The antiviral signal was consistently validated using western blotting, qRT-PCR, plaque assays, and microscopy, which bolstered the central argument. However, the scope of this study was restricted to a single seasonal/common coronavirus model and in vitro assays, necessitating a cautious approach to translational claims.

-> Thank you for your insightful comment. We agree that further studies using additional coronavirus models and in vivo systems are necessary to confirm the broader antiviral potential of our findings. However, HCoV-OC43 belongs to the genus *Betacoronavirus*, which also includes highly pathogenic coronaviruses such as SARS-CoV-2 and MERS-CoV. Therefore, HCoV-OC43 is a useful and relatively safe model for investigating coronavirus replication and evaluating potential antiviral compounds. Furthermore, our findings were consistently supported by multiple experimental approaches, including Western blotting, qRT-PCR, plaque assays, and microscopy. Therefore, we believe that this study provides valuable preliminary evidence supporting the antiviral potential of *Rumex crispus*.

The primary shortcoming of this study is its failure to convincingly demonstrate that emodin is the exclusive or primary factor responsible for the activity in the active fractions. The active fraction may result from synergy, co-enrichment of related anthraquinones, or the extraction of various bioactive components. The manuscript currently overemphasizes the notion that "emodin is the principal contributor" without providing sufficient mechanistic evidence. The fraction requires adequate attention to claim the novelty of the work from the existing ones.

🡪 Thank you for your insightful and constructive comments. We agree that the antiviral activity of the active fractions cannot be attributed exclusively to emodin, as other bioactive constituents and potential synergistic effects may also contribute to the observed activity.

As recommended, we have revised the Abstract, Results, and Discussion sections to avoid overstating the contribution of emodin as the principal antiviral component. We have also expanded the Discussion to acknowledge these limitations and the need for further studies to clarify the roles of individual compounds and their potential synergistic effects.

We emphasize that the novelty of our study lies in demonstrating the antiviral activity of *R. crispus* fractions against HCoV-OC43 and identifying emodin as one of the compounds exhibiting antiviral activity.

- Abstract: “These findings suggest that emodin may contribute to the antiviral activity of *R. crispus* extract, although the potential involvement of other bioactive constituents cannot be excluded.”

- Results: “These results suggest that emodin may be one of the active compounds contributing to the inhibitory effects of *R. crispus* extract on coronavirus replication.”

- Discussion: “In this study, we demonstrated that root extracts of *R. crispus* exhibit antiviral activity against human coronavirus, to which the bioactive anthraquinone emodin may contribute, along with other bioactive constituents.”

The study predominantly focused on HCoV-OC43, yet it failed to consider whether the results might be applicable to the more clinically significant SARS-CoV-2 or other human coronaviruses.

-> Thank you for your valuable comment. As recommended by the reviewer, we have revised the Introduction to clarify the rationale for selecting HCoV-OC43 and to discuss the potential relevance of our findings to other human coronaviruses, including SARS-CoV-2 and MERS-CoV.

“In addition to SARS-CoV-2, which caused the recent COVID-19 pandemic, several human coronaviruses, including HCoV-OC43, HCoV-229E, HCoV-NL63, and HCoV-HKU1, are responsible for a substantial proportion of common cold infections [7, 8]. Among these viruses, HCoV-OC43 has attracted considerable attention because it belongs to the genus *Betacoronavirus*, which also includes SARS-CoV-2 and MERS-CoV [9]. Due to its phylogenetic relationship with these highly pathogenic coronaviruses and its relatively low pathogenicity, HCoV-OC43 has been used as a surrogate model for the initial screening and evaluation of potential antiviral agents [10]. Therefore, investigating antiviral compounds against HCoV-OC43 may provide valuable insights into the development of therapeutic candidates targeting human coronaviruses, including SARS-CoV-2 [11].”

The statistical method employed is inadequate and simplistic, as it relies on repeated two-group t-tests in Excel, which are unsuitable for handling the various concentrations and conditions. Additionally, the manuscript lacks strong control for multiple comparisons and does not provide a comprehensive dose–response model.

-> Thank you for your valuable comments. As recommended by the reviewer, we have revised our statistical analysis methods to improve the robustness and reliability of our results. Specifically, we reanalyzed the data using one-way ANOVA followed by Dunnett’s multiple comparisons test in GraphPad Prism software to appropriately account for multiple comparisons among treatment groups and the control group. Accordingly, the statistical analyses presented in Figures 1, 2, 3, and 7 have been updated in the revised manuscript.

In addition, dose–response curves were generated using nonlinear regression analysis with a four-parameter logistic (4PL) model to estimate the IC50 values.

Furthermore, language and editing problems in the manuscript diminish clarity and occasionally complicate the understanding of mechanistic interpretation.

-> Thank you for your comment. We have carefully revised the manuscript to improve its clarity, grammar, and scientific accuracy.

Reviewer #2:

Title: Rumex crispus Extract Exhibits Antiviral Activity Against Coronavirus and Contains the Active Compound Emodin
Number : PONE-D-26-16257
Cho S. et al. present antiviral effects of extracts obtained from Rumex crispus and isolate Emodin as active compound.
The authors should explain why they chose HCoV-OC43 for this study, and consider whether the results can be applied to other coronaviruses — particularly highly virulent ones as SARS-CoV-2 or MERS-CoV.

-> Thank you for your valuable comment. As recommended by the reviewer, we have revised the Introduction to clarify the rationale for selecting HCoV-OC43 and to discuss the potential relevance of our findings to other human coronaviruses, including SARS-CoV-2 and MERS-CoV.

“In addition to SARS-CoV-2, which caused the recent COVID-19 pandemic, several human coronaviruses, including HCoV-OC43, HCoV-229E, HCoV-NL63, and HCoV-HKU1, are responsible for a substantial proportion of common cold infections [7, 8]. Among these viruses, HCoV-OC43 has attracted considerable attention because it belongs to the genus *Betacoronavirus*, which also includes SARS-CoV-2 and MERS-CoV [9]. Due to its phylogenetic relationship with these highly pathogenic coronaviruses and its relatively low pathogenicity, HCoV-OC43 has been used as a surrogate model for the initial screening and evaluation of potential antiviral agents [10]. Therefore, investigating antiviral compounds against HCoV-OC43 may provide valuable insights into the development of therapeutic candidates targeting human coronaviruses, including SARS-CoV-2 [11].”

Result section
The authors referred to “conditioned media”. Could the authors explain this concept, please? If it related to cell culture supernatants, it would be clearer to use that term.

-> Thank you for your comment. As recommended by the reviewer, we have replaced the term “conditioned media” with “cell culture supernatants” throughout the manuscript.

Could the authors, please, provided a detailed description of the method used to obtain the results for “relative RNA expression level” (figure 3)?

-> Thank you for your comment. As recommended by the reviewer, we have provided additional details in the Materials and Methods section of the revised manuscript, as shown below.

“To quantify coronavirus RNA levels in both cells and cell culture supernatants, quantitative RT-PCR was performed as described in a previous report [9]. In brief, total RNA was isolated from harvested cells and cell culture supernatants using PURE™ Total RNA Extraction Kit (Infusion Tech, Anyang, Korea), following the manufacturer’s guidelines. For the quantification of viral RNA in cell culture supernatants, total cellular RNA was added as carrier RNA. Complementary DNA (cDNA) was synthesized from equal amounts of total RNA using the M-MLV cDNA Synthesis Kit (Enzynomics, Daejeon, Republic of Korea). Quantitative real-time PCR (qRT-PCR) was subsequently carried out on a QuantStudio 3 Real-Time PCR System (Applied Biosystems, Thermo Fisher Scientific, Waltham, MA, USA) with 2× Real-Time PCR Master Mix containing SYBR Green (BioFACT, Daejeon, Republic of Korea). RPL4 gene expression was used for normalization.”

The same applies to the results relating to protein expression levels (Figure 1).

-> Thank you for your comment. As recommended by the reviewer, we have provided additional details in the Materials and Methods section of the revised manuscript, as shown below.

“HCoV-OC43–infected RD cells and cell culture supernatants were collected separately. Cells were lysed in lysis buffer (150 mM NaCl, 50 mM HEPES at pH 7.5, and 1% NP-40) supplemented with a protease inhibitor cocktail (Roche, Basel, Switzerland). Equal amounts of cell lysates (20 µg of total protein) and equal volumes of cell culture supernatants (20 µl) were subjected to SDS-PAGE and analyzed by Western blotting using an anti-HCoV-OC43 antibody. Mouse polyclonal anti-HCoV-OC43 antibodies were generated using the inactivated HCoV-OC43 particles in mice, as described previously [25]. Protein bands were visualized using the ChemiDoc™ Imaging System (Bio-Rad Laboratories, Hercules, CA, USA). HCoV-OC43 protein levels in cell lysates were normalized to GAPDH expression.”

Could the authors, please, explain how the concentrations of plant extracts and emodin tested in the study were selected?

-> Thank you for your comment. The concentrations of the plant extracts and emodin used in this study were selected based on preliminary experiments evaluating their cytotoxicity and antiviral activity. First, we assessed the cytotoxicity of these compounds to determine the concentration ranges that did not significantly affect host cell viability. Next, we evaluated their antiviral activities to determine appropriate concentration ranges for IC50 calculation. As shown in Figs. 1–3, the plant extracts did not significantly affect cell viability at concentrations up to 80 µg/mL, while the highest IC50 value was approximately 29 µg/mL. Therefore, we selected concentrations up to 40 µg/mL for subsequent antiviral experiments to ensure sufficient antiviral activity without significant cytotoxicity. Similarly, emodin concentrations up to 20 µM were selected based on its cytotoxicity and antiviral activity profiles, as shown in Fig. 7C.

Why was the cytotoxicity of emodin alone not tested?

-> Thank you for your comment. As recommended, we have added the cytotoxicity data for emodin to the revised manuscript. As shown in Fig. 7C, emodin treatment at concentrations up to 20 µM did not significantly affect cell viability.
